# Predicting progression of cognitive decline to dementia using dyadic patterns of subjective reporting: evidence from the CompAS longitudinal study

**DOI:** 10.3389/fnagi.2024.1319743

**Published:** 2024-02-02

**Authors:** Lucía Pérez-Blanco, Alba Felpete-López, Ana Nieto-Vieites, Cristina Lojo-Seoane, María Campos-Magdaleno, Fátima Fernández-Feijoo, Onésimo Juncos-Rabadán, Arturo X. Pereiro

**Affiliations:** ^1^Department of Developmental Psychology, Faculty of Psychology, Universidade de Santiago de Compostela, Santiago de Compostela, Spain; ^2^Applied Cognitive Neuroscience and Psychogerontology Group, Health Research Institute of Santiago (IDIS), Universidade de Santiago de Compostela, Santiago de Compostela, Spain

**Keywords:** subjective cognitive complaints, self-report, informant-report, depressive symptomatology, subjective cognitive decline, mild cognitive impairment, dementia

## Abstract

**Objective:**

To analyze the validity of self and informant reports, depressive symptomatology, and some sociodemographic variables to predict the risk of cognitive decline at different follow-up times.

**Methods:**

A total of 337 participants over 50 years of age included in the CompAS and classified as Cognitively Unimpaired (CU), Subjective Cognitive Decline (SCD) and Mild Cognitive Impairment (MCI) groups were assessed at baseline and three follow-ups. A short version of the QAM was administered to assess the severity of subjective cognitive complaints (SCCs), and the GDS-15 was used to evaluate the depressive symptoms. At each follow-up assessment, participants were reclassified according to the stability, regression or progression of their conditions. Logistic regression analysis was used to predict which CU, SCD and MCI participants would remain stable, regress or progress at a 3rd follow-up by using self- and informant-reported complaints, depressive symptomatology, age and education at baseline and 2nd follow-ups as the predictive variables.

**Results:**

Overall, self-reported complaints predicted progression between the asymptomatic and presymptomatic stages. As the objective deterioration increased, i.e., when SCD progressed to MCI or dementia, the SCCs reported by informants proved the best predictors of progression. Depressive symptomatology was also a predictor of progression from CU to SCD and from SCD to MCI.

**Conclusion:**

A late increase in self-reported complaints make valid estimates to predict subjective decline at asymptomatic stages. However, an early increase in complaints reported by informants was more accurate in predicting objective decline from asymptomatic stages. Both, early and late decrease in self-reported complaints successfully predict dementia from prodromic stage. Only late decrease in self-reported complaints predict reversion from prodromic and pre-symptomatic stages.

## 1 Introduction

Subjective Cognitive Decline (SCD) refers to a self-reported cognitive decline relative to the functioning previously perceived in the absence of objective cognitive impairment and unrelated to an acute event (Jessen et al., 2014). Cognitive complaints may be experienced along the continuum of cognitive decline from normative aging to dementia (Rabin et al., 2015), through preclinical stages (Jessen et al., 2020) to presymptomatic and/or prodromal stages of dementia, particularly Alzheime’s disease (AD) (Jack et al., 2018). Such complaints are considered the first obvious manifestation of the earliest preclinical phase of AD and have been associated with AD biomarkers such as amyloid and tau proteins (Jack et al., 2018; Janssen et al., 2022; Jessen et al., 2023). Some researchers refer to cognitive complaints as a possible neurobehavioral expression of the underlying neurodegenerative process (Snitz et al., 2015). Personal factors such as mood disorders have also been associated with cognitive complaints (Comijs et al., 2002; Burmester et al., 2016; Markova et al., 2017; Tandetnik et al., 2017), and specifically depressive symptoms have been associated with SCD (Zlatar et al., 2018; Ryu et al., 2020; Zullo et al., 2021; Kim and Lee, 2022). Depressive symptoms have been also shown to be one of the main predictors of progression from SCD to MCI and/or dementia (Dolcet-Negre et al., 2023).

Self-reported complaints are currently a core criterion in the diagnosis of SCD and Mild Cognitive Impairment (MCI), whereas informant-reported complaints have been considered simply as additional evidence of or risk factor for progression to MCI or conversion to dementia (Petersen, 2004; Dubois et al., 2007; Albert et al., 2011; Jessen et al., 2014). Study of the value of the patient’s report and that of the corresponding informant in predicting cognitive decline is an important topic in current research using two types of analysis: (a) awareness for comparison between self- and informant-reported measures or comparison between self-reported and objective measures; and (b) separate analysis of the self- and informant-reported measures. Some authors have used the Awareness of Cognitive Decline (ACD) concept, operationalized as the difference between the patient’s complaints and the informant’s complaints on a memory complaint scale (Cacciamani et al., 2020; Ryu et al., 2020; Vannini et al., 2020), or as the residual obtained from regressing the objective memory performance score against a subjective complaint score (the deviation of the objective performance from their subjective rating) (Munro et al., 2018). In a population of individuals with memory complaints, ACD did not predict cognitive impairment measured by different neuropsychological tests; however, individuals with low ACD had a higher amyloid burden than those with normal ACD and therefore had a higher risk of developing AD (Cacciamani et al., 2020). In a cross-sectional study, Ryu et al. (2020) compared ACD in healthy controls, SCD, MCI and very mild AD. These researchers found no differences between self-reported and informant-reported complaints in the healthy controls group, but positive discrepancy scores (i.e., self > informant) in the SCD group, and negative scores (i.e., self < informant) in the MCI group. In addition, between-group comparisons of discrepancy scores showed higher scores in SCD than in the MCI group, suggesting decreased awareness in MCI. Another study found that participants with MCI who progressed to AD had lower awareness of their condition than participants with stable MCI and showed that baseline awareness predicts progression to AD (Munro et al., 2018).

Other authors have used self- and informant-reported measures separately in their studies. A recent meta-analytic study (Perez-Blanco et al., 2022) found that reports of SCCs, especially from informants, were associated with an increased risk of progression from CU to MCI and/or dementia. The increasing likelihood of misreporting cognitive decline in the later stages of disease progression highlights the importance of informant reports in predicting cognitive decline (Nosheny et al., 2022). This recent narrative review by a working group in The Subjective Cognitive Decline Professional Interest Area within the Alzheimer’s Association ISTAART also found that evidence for the validity of dyad-reported measures is inconclusive (Nosheny et al., 2022). Whereas the findings at the MCI stage indicate that informant-reported complaints would better identify the risk of dementia than self-reported complaints, research with CU participants shows that both self- and informant-reported measures predict the risk of cognitive decline, and no research has been reported with SCD participants. The review also highlights that other external factors, such as sociocultural conditions, may contribute to the risk. A recent study by Numbers et al. (2023) examined longitudinal changes in patient and informant reports of SCC to determine whether the SCCs were associated with increased risk of incident dementia. Their findings indicate that at the beginning of the decline process, the patients were more likely to report SCCs than the informants, whereas the informants became more likely to report SCCs over time, suggesting that this change appears to be uniquely prognostic of future dementia.

To date, and despite the clinical relevance of SCCs in the early or later stages of dementia, we are not aware of any study that has analyzed the value of SCCs for predicting the progression of cognitive impairment through the different stages of cognitive continuum (CU, SCD, MCI and dementia), considering different times at which the complaints are reported. Therefore, the main objective of this study was to independently analyze the value of self- and informant-reported SCCs, while also considering the influence of depressive symptomatology and sociocultural factors, such as age and education, as predictors of risk of cognitive decline in CU, SCD and MCI participants, measured at two-time points of the longitudinal assessment: at an early stage (i.e., measured at baseline, 54–72 months before the end of the follow-up) and at a later stage (i.e., measured at 2nd follow-up, 18–24 months before the end of the follow-up).

## 2 Materials and methods

### 2.1 Participants

The initial sample comprised of 435 volunteers, aged 50 or over, enrolled in the Compostela Aging Study (CompAS). The CompAS is an ongoing longitudinal project initiated in 2008 and aimed at the early detection and progression of cognitive impairment in patients with SCCs aged over 50 years attending Primary Care Health Centers in Galicia (north-west Spain) (Juncos-Rabadán et al., 2014). The CompAS is currently composed of three cohorts, and the participants in the present study belong to the first cohort. Patients who met any of the following criteria were excluded from the study: prior diagnosis of major psychiatric illness, prior diagnosis of neurological disease, including probable AD or other types of dementia; previous brain damage or brain surgery; previous chemotherapy treatment; prior diagnosis of diabetes type II; sensory or motor disturbances that might affect the normal performance of the tasks; or no prior drug consumption including alcohol.

All participants volunteered to participate in the study and provided written informed consent. The CompAS has been approved by the Galician Autonomous Committee on Research Ethics (Xunta de Galicia, Spain) and developed under the provisions of the Helsinki Declaration and revised in Seoul (2008).

### 2.2 Neuropsychological assessment

The participants underwent clinical, neurological and neuropsychological evaluations, conducted, respectively, by general practitioners, cognitive neurologists and psychologists who are experts in aging and dementia. The comprehensive neuropsychological battery of tests used to diagnose the participants was composed of the following: (a) an *ad hoc* Sociodemographic and Health Questionnaire, to gather personal, educational and medical information; (b) the Mini-Mental State Examination (MMSE) test, to assess a global cognitive performance (Folstein et al., 1975; Spanish version Lobo et al., 1999); (c) the California Verbal Learning Test (CVLT), to assess objective memory (Delis et al., 1987; Spanish version Benedet and Alejandre, 1998); (d) the Cambridge Cognitive Assessment Revised (CAMCOG-R), to evaluate cognitive performance by domains, orientation, language, memory, attention/calculation, praxis, abstract thinking, perception and executive functions (Roth et al., 1986; Spanish version López-Pousa, 2003; age and education norms by Pereiro et al., 2015); (e) the “Lawton and Brody’s Scale for Instrumental Activities of Daily Living,” to determine the degree of functionality (Lawton and Brody, 1969; Spanish version Vergara et al., 2012); and (f) the short form of the Geriatric Depression Scale (GDS-15), to evaluate depressive symptomatology (Sheikh and Yesavage, 1986; Spanish validation of Martínez de la Iglesia et al., 2002).

A short version of the Questionnaire d’ auto-évaluation de la Mémoire (QAM) (Van der Linden et al., 1989; Benedet and Seisdedos, 1996) was administered to participants and their respective informants to evaluate SCCs. This version assesses the frequency of forgetfulness, distraction, difficulties in lexical access, and spatial orientation. It comprises 7 items each scored on a Likert scale ranging from 1 to 5 (“never,” “rarely,” “sometimes,” “often” and “always”). It includes the following items: (1) How often do you forget where you left your things?”; (2) “How often do you forget the names of people you just met?”; (3) “How often do you forget the names of close relatives or friends?”; (4) “How often do you have a word on the tip of your tongue?”; (5) “How often do you find yourself lost in familiar places where you have been before?”; (6) “How often do you find yourself lost in unfamiliar places where you have been a few times?”; and (7) “How often do you forget things you planned to do?.” The reliability of this QAM short version, tested in participants from the first cohort of the CompAS, was 0.69 (Cronbach’s alpha) for patient score and 0.78 for informant scoring (Rivas-Fernández et al., 2023). The cut-off point, which corresponds to the 5th percentile of the total QAM short version scoring adjusted for age, has been shown to be a valid measure of SCCs for predicting progression to MCI and dementia (Pereiro et al., 2021).

### 2.3 Clinical diagnoses

Considering the clinical, neurological, and neuropsychological evaluations, diagnoses were reached by consensus at special meetings held by the research team. Participants were diagnosed (CU, SCD, MCI or dementia) at baseline and re-diagnosed at the 1st, the 2nd^,^ and 3rd follow-ups with a follow-up interval of 18–24 months.

Diagnosis of SCD was performed following the two main criteria described by the SCD-initiative (SCD-I) Working Group on Alzheimer’s Dementia (Jessen et al., 2014, 2020): (1) self-perceived persistent decline in cognition, particularly in memory, relative to previously normal cognitive status, that is unrelated to an acute event; and (2) normal performance in cognitive tests used to classify MCI tests adjusted for age and education. For the first criterion, the participants were asked if they perceived difficulties relative to attention or memory that concerned them in the last few years, and the informants were asked for confirmation of any such difficulties. In addition, considering that complaints characterize the normal aging process (Rabin et al., 2015; Markova et al., 2017), SCCs were considered beyond the normative threshold when the QAM score (patient) was above the cut-off point corresponding to the age-adjusted 5th percentile according to norms proposed by Pereiro et al. (2021).

Participants were categorized as CU when they did not fulfil the previous SCD criteria even though they reported SCCs that did not exceed the 5th percentile in the QAM and their overall cognitive performance was within the normal range for age and educational level.

Diagnosis of MCI was based on the consensus criteria recommendations of the National Institute on Aging-Alzheimer’s Association workgroups (Petersen, 2004; Winblad et al., 2004; Albert et al., 2011): (a) evidence of concern regarding a change in cognition corroborated by the patient and the informant (measured through QAM); (b) objective evidence of impairment in one or more cognitive domains that are greater than expected for the patient’s age and educational background. This criterion was considered fulfilled when the scores were in the 1–2 standard deviation range (between the 3rd and 16th percentiles) below the norm by age and education; (c) preservation or minimal affectation in instrumental activities of daily living tested by the Lawton and Brody Index (Lawton and Brody, 1969); and (d) no diagnosis of dementia defined by NINCDS-ADRDA (Dubois et al., 2007) and DSM-IV criteria (APA, 1994).

Dementia was diagnosed according to DSM-IV-TR criteria (American Psychiatric Association, 2000): (a) objective evidence of impairment in memory, and in other cognitive domains, that is greater than expected for the patient’s age and educational background. This criterion was met when the scores in the corresponding neuropsychological tests were below 2 standard deviations according to the norms by age and education; (b) a gradual onset and continued cognitive decline; (c) cognitive deficits are not due to other neurological or systemic diseases, nor are they induced by substances; (d) deficits do not appear exclusively during the course of delirium; (e) the alteration is not better explained by the presence of other affective disorders; and (f) severe effects in instrumental activities of daily living tested by the Lawton and Brody Index (Lawton and Brody, 1969).

**Figure 1:**
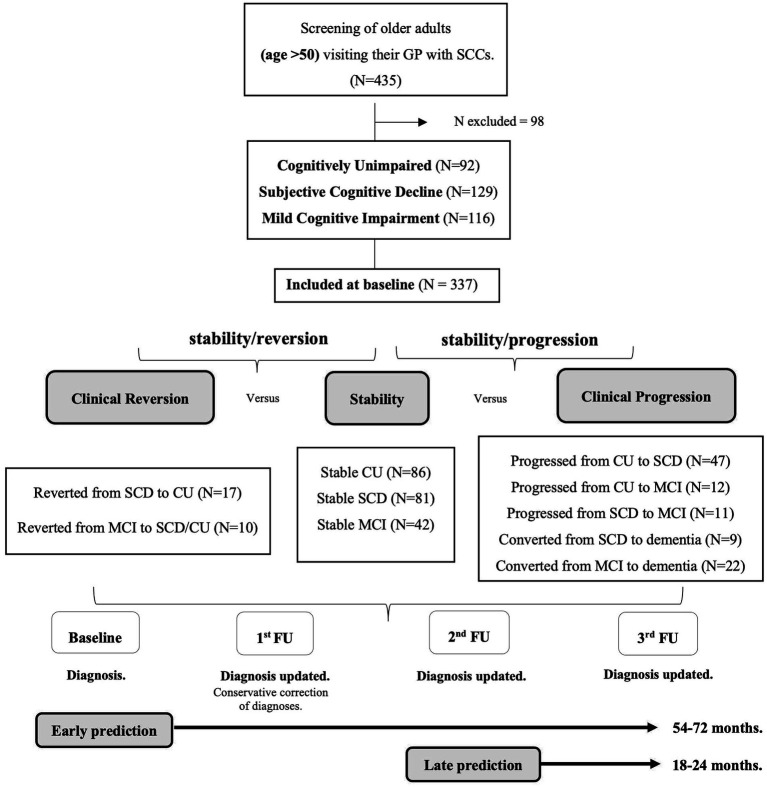
Flowchart of the grouping process according to the stability, reversion, or progression patterns. Notes: SCCs = Subjective Cognitive Complaints; GP = General Practitioner; CU = Cognitively Unimpaired; SCD = Subjective Cognitive Decline; MCI = Mild Cognitive Impairment; FU = Follow-Up.

The diagnosis at baseline was conservatively adjusted when diagnosis at the first follow-up (18–24 months after baseline) showed an unexpected cognitive recovery (i.e., from MCI to SCD and/or CU; from SCD to CU) (Pereiro et al., 2021). However, participants who had improved at the second or third follow-up were included in two groups that reverted to their baseline diagnosed status, i.e., from SCD to CU or from MCI to SCD and/or CU, respectively. Participants who were not evaluated twice throughout the follow-up were excluded from furhter study (*N* = 98, 22.52% of the initial sample). Subsequently, at each follow-up assessment, participants diagnosed as CU, SCD, MCI or dementia were reclassified according to stability of their condition (i.e., diagnosis remains unchanged from baseline to the third follow-up), or a change in diagnosis at some point between the baseline and the third follow-up (i.e., negative changes when they get worse or progress, or positive changes when they improve or revert). Progression or reversion were always determined considering the diagnosis at the end of the entire evaluation process and were classified into various patterns that defined five groups of progression and two groups of reversion. The groups in which cognitive decline progressed were as follows: (1) participants who progressed from CU to SCD; (2) participants who progressed from CU to MCI; (3) participants with SCD who progressed to MCI; (4) participants with SCD who progressed to dementia, and (5) participants with MCI who progressed to dementia. The two groups established according to reversion (clinical improvement) were as follows: (1) participants who reverted from MCI to SCD and/or CU, and (2) participants with SCD who reverted to CU. The pattern of progression from CU to dementia was not considered because no such cases were observed. The final sample consisted of 337 participants. See the flowchart of the grouping process in Figure 1.

### 2.4 Statistical analyses

Group differences in socio-demographic, subjective reporting (self and informant) and depressive symptomatology of the cognitive regression/progression groups at baseline were analyzed by Kruskal-Wallis tests for continuous variables and *χ^2^* tests for categorical variables. The Kolmogorov–Smirnov test was applied to test for normality distribution.

Binary logistic regression analysis was used to investigate the value of cognitive complaints (i.e., self-reported/informant-reported SCCs) and depression symptoms assessed at baseline (i.e., early prediction) and 2nd follow-up (i.e., late prediction) to predict (1a) progression in cognitive decline at 3rd follow-up for the CU, SCD, and MCI stages or (1b) cognitive regression or cognitive improvement for the SCD and MCI stages. The effects of age and education were also controlled, and their relationship with diagnosis of cognitive decline was considered.

Age, years of schooling, self-reported SCCs, informant-reported SCCs, and depressive symptomatology were selected for multiple regression as predictor variables using the enter method.

First, separate binary logistic regression models were used to test the association between predictive variables and the outcome (i.e., progression/stable) according to the following transition patterns: (a) progressing cases, which correspond to participants in the group CU who progressed to SCD, and stable cases which correspond to CU participants that do not change; (b) progressing cases, which correspond to participants in the CU group who progressed to MCI, and stable cases which correspond to the stable-CU group; (c) progressing cases, which correspond to participants in the group SCD who progressed to MCI, and stable cases which correspond to participants in the stable-SCD group; (d) progressing cases, which correspond to participants in the SCD group who converted to dementia, and stable cases which correspond to participants in the stable-SCD group; and (e) progressing cases, which correspond to participants in the MCI group who converted to dementia, and stable cases which correspond to participants in the stable-MCI group.

Secondly, separate binary logistic regression models were used to test the association between predictive variables and the outcome (i. e., reversion/stable) according to the following patterns of clinical recovery: a) reversion cases, which correspond to participants belonging to the group SCD who reverted to CU, and stable cases which correspond to those in the stable-SCD group; and b) reversion cases, which correspond to MCI participants who reverted to SCD and/or CU, and stable cases which correspond to participants in the stable-MCI group.

The missing data for informant scores were replaced with the mean score of the corresponding diagnostic group following the missing case imputation method (*N* = 20 for CU group, *N* = 18 for SCD group, *N* = 24 for MCI group; total *N* = 62, 18.39% out of the total sample). All statistical analyses were performed using SPSS version 27.0.0 (SPPS Inc., Chicago, IL, United States). A probability level of *p* < 0.05 was considered to indicate statistical significance.

## 3 Results

Descriptive statistics and between-group comparisons in age, sex, education, self- and informant- reported complaints for stable and progression groups are summarized in Table 1. Descriptive statistics and comparison for the groups that improved in terms of reversion of their cognitive status are shown in Table 2. The most frequent relationships between participants and informants in all groups, i.e., spouses and descendants, are also shown in Tables 1, 2. Comparison between diagnostic groups revealed similar profiles in sociodemographic and predictive variables. Informant-reported SCCs and depression were significantly higher in the group that progressed from CU to SCD than in the stable CU group. No significant differences in these parameters were observed in the comparison of the stable CU group and the CU group who progressed to MCI. The SCD group that progressed to MCI group had significantly fewer years of education than the stable SCD group. The SCD group that converted to dementia was significantly older than the stable SCD group. The MCI group that progressed to dementia was significantly older and had a significantly lower level of self-reported SCCs. Finally, the MCI group that reverted to CU and/or SCD group was significantly younger than the MCI stable group.

**Table 1:** Descriptive characteristics of the participants at baseline in the groups of patients undergoing progression in cognitive decline and their informants.

	PROGRESSION OF COGNITIVE DECLINE												
Variables	Stable CU (*n* = 86)	CU progression to SCD (*n* = 47)	Group difference (a) Final model	CU progression to MCI (*n* = 12)	Group difference (b) Final model	Stable SCD (*n* = 81)	SCD progression to MCI (*n* = 11)	Group difference (c) Final model	SCD progression to dementia	Group difference (d) Final model	Stable MCI (*n* = 42)	MCI progression to dementia (*n* = 22)	Group difference (e) Final model
Age (in years)	64.54 (8.79)	63.57 (8.95)	***K-W of H*** = 0.186 ***p*** = 0.666	68.08 (9.82)	***K-W of H*** = 1.515 ***p*** = 0.218	66.09 (8.3)	69.63 (8.82)	***K-W of H*** = 1.695 ***p*** *= 0*.193	71.66 (3.00)	***K-W of H*** = 5.092 ***p*** = 0.024*	71.83 (7.33)	75.50 (5.82)	***K-W of H*** = 3.902 ***p*** = 0.048*
Gender (%Women)	66.3%	61.7%	**χ2** = 0.279 ***p*** = 0.598	41.7%	**χ2** = 2.745 ***p*** = 0.098	76.5%	90.9%	**χ2** = 1.175 ***p*** = 0.278	66.7%	**χ2** = 0.428 ***p*** = 0.513	69.0%	45.5%	**χ2** = 3.376 ***p*** = 0.066
Education (in years)	10.58 (4.81)	10.44 (4.82)	***K-W of H*** = 0.022 ***p*** = 0.882	9.25 (4.02)	***K-W of H*** = 1.06 ***p*** = 0.293	9.39 (4.02)	6.54 (2.94)	***K-W of H*** = 5.221 ***p*** = 0.022*	8.55 (3.90)	***K-W of H*** = 0.367 ***p*** = 0.545	9.33 (3.99)	9.81 (5.09)	***K-W of H*** = 0.004 ***p*** = 0.949
Self-reported SCCs	16.67 (3.54)	17.27 (3.37)	***K-W of H*** = 1.266 ***p*** = 0.260	15.41 (4.75)	***K-W of H*** = 2.132 ***p*** = 0.144	21.70 (3.77)	21.45 (3.29)	***K-W of H*** = 0.005 ***p*** = 0.942	19.77 (3.59)	***K-W of H*** = 3.190 ***p*** = 0.074	20.42 (4.75)	17.72 (3.39)	***K-W of H*** = 5.611 ***p*** = 0.018*
Informant-reported SCCs	14.29 (3.85)	16.38 (3.41)	***K-W of H*** = 10.97 ***p*** < 0.001**	15.83 (3.56)	***K-W of H*** = 2.911 ***p*** = 0.088	16.58 (4.40)	17.36 (5.06)	***K-W of H*** = 0.040 ***p*** = 0.841	17.33 (2.34)	***K-W of H*** = 1.948 ***p*** = 0.163	17.31 (4.00)	18.77 (3.57)	***K-W of H*** = 0.678 ***p*** = 0.410
Depressive symptoms	2.55 (2.46)	3.72 (2.81)	***K-W of H*** = 6.715 ***p*** = 0.010*	3.16 (2.32)	***K-W of H*** = 1.344 ***p*** = 0.246	4.24 (3.17)	5.45 (3.47)	***K-W of H*** = 1.334 ***p*** = 0.248	2.44 (1.58)	***K-W of H*** = 2.691 ***p*** = 0.101	3.61 (2.83)	2.86 (2.74)	***K-W of H*** = 1.540 ***p*** = 0.215
**Relationship with the INFORMANTS**													
Spouse	54.4%	61.1%		63.6%		45.6%	12.5%		88.9%		44.1%	47.1%	
Descendants	42.6%	38.9%		36.4%		51.5%	75%		11.1%		47.1%	29.4%	
Others	1.5%	–		–		2.9%	12.5%		–		8.8%	5.9%	

CU, cognitively unimpaired; SCD, subjective cognitive decline; MCI, mild cognitive impairment; (a) Stable CU vs. CU progressed to SCD; (b) Stable CU vs. CU progressed to MCI; (c) Stable SCD vs. SCD progressed to MCI; (d) Stable SCD vs. SCD converted to dementia; (e) Stable MCI vs. MCI converted to dementia; K-W of H, Kruskal-Wallis of H; χ2, Pearson’s chi statistic. Group statistics based on Kruskal-Wallis-tests for continuous variables and χ2 tests for categorical variables. Significance level *p < 0.05; **p < 0.001.

**Table 2:** Descriptive characteristics at baseline of the participants who reverted to CU and/or SCD, and their informants.

	REGRESSION OF COGNITIVE DECLINE					
Variables	Stable SCD (*n* = 81)	SCD reversion to CU (*n* = 10)	Group difference (a) Final model	Stable MCI (*n* = 42)	MCI reversion to CU and/or SCD (*n* = 17)	Group difference (b) Final model
Age (in years)	66.09 (8.3)	60.30 (9.53)	***K-W of H*** = 3.465 ***p*** = 0.063	71.83 (7.33)	66.64 (7.06)	***K-W of H*** = 6.540 ***p*** = 0.011*
Gender (%Women)	76.5%	80.0%	**χ2** = 0.060 ***p*** = 0.807	69.0%	70.6%	**χ2** = 0.014 ***p*** = 0.907
Education (in years)	9.39 (4.02)	12.00 (5.41)	***K-W of H*** = 2.780 ***p*** = 0.095	9.33 (3.99)	8.00 (2.97)	***K-W of H*** = 1.251 ***p*** = 0.263
Self-reported SCCs	21.70 (3.77)	20.70 (2.26)	***K-W of H*** = 0.288 ***p*** = 0.592	20.42 (4.75)	19.00 (1.78)	***K-W of H*** = 0.691 ***p*** = 0.406
Informant-reported SCCs	16.58 (4.40)	16.80 (3.64)	***K-W of H*** = 0.083 ***p = ***0.774	17.31 (4.00)	15.82 (3.55)	***K-W of H*** = 1.955 ***p*** = 0.162
Depressive symptoms	4.24 (3.17)	5.10 (2.92)	***K-W of H*** = 0.862 ***p*** = 0.353	3.61 (2.83)	3.58 (2.85)	***K-W of H*** = 0.006 ***p*** = 0.939
**Relationship with the INFORMANTS**						
Spouse	45.6%	37.5%		44.1%	30.8%	
Descendants	51.5%	50.0%		47.1%	46.2%	
Others	2.9%	12.5%		8.8%	23.1%	

CU, cognitively unimpaired; SCD, subjective cognitive decline; MCI, mild cognitive impairment; (a) stable SCD vs. SCD reverted to CU; (b) Stable MCI vs. MCI reverted to CU and/or SCD. K-W of H, Kruskal-Wallis of H; χ2, Pearson’s chi statistic. Group statistics based on Kruskal-Wallis-tests for continuous variables and χ2 tests for categorical variables. Significance level *p < 0.05; **p < 0.001.

Correlations between the main predictor variables were low-moderate, and the highest value was always below 0.50 (i.e., Self-reported depression = 0.41, Self-reported depression = 0.41, *p* < 0.001 in SCD; Self-reported depression = 0.48, *p* < 0.001 in MCI), indicating an absence of multicollinearity.

### 3.1 Predicting progression in the CU group

In the CU group, progression to SCD was significantly associated with higher informant-reported SCCs (*β* = 0.154, *SE* = 0.060, *p* = 0.010, *OR* = 1.167, *CI* = 1.038–1.311), for early prediction, and with higher self-reported SCCs (*β* = 0.356, *SE* = 0.091, *p* = 0.001, *OR* = 1.428, *CI* = 1.428–1.194) and higher depressive symptomatology (*β* = 0.193, *SE* = 0.095, *p* = 0.043, *OR* = 1.212, *CI* = 1.006–1.461) for late prediction (see Table 3). The model fitting parameters (*R*^2^ and HL) indicated acceptable fits, but with low values, with slightly better fits for late prediction than for early prediction. Classification values were also low, and they were better for late prediction (Specificity =80.4, Sensitivity = 69.8 Overall percentage = 75.8) than for early prediction (Specificity =88.4, Sensitivity = 29.4, Overall percentage = 67.7). Progression to MCI was only significantly associated with older age (*β* = 0.091, *SE* = 0.045, *p* = 0.041, *OR* = 1.096, *CI* = 1.004–1.196) for late prediction. The model fits were acceptable, and the classification values for Sensitivity were very low (8.3 for early prediction and 18.2 for late prediction).

**Table 3:** Prediction of the risk of progression/conversion according to the five patterns of progression relative to stability.

	EARLY PREDICTION Time in months: M 58.87; SD 8.26 –From baseline to the 3rd FU–							LATE PREDICTION Time in months: M 21.60; SD 7.36 –From the 2nd FU the 3rd FU –						
**(1) CU to SCD**	No cases, **STABLE CU**: 86; Cases, **progressed to SCD**: 47							No cases, **STABLE CU**: 56; Cases, **progressed to SCD**: 43						
	***β***	***S.E.***	***Wald***	***p***	***OR***	***95% CI***	***Models***	***β***	***S.E.***	***Wald***	***p***	***OR***	***95%CI***	***Models***
Age	−0.013	0.023	0.331	0.565	0.987	0.944–1.032	**N of *R***^**2**^: 0.136 **H-L:** 5.618 ***p*** = 0.690 **SP** = 88.4 **SE** = 29.8 **Ov** = 67.7	0.026	0.032	0.657	0.418	1.026	0.964–1.093	**N of *R***^**2**^: 0.362 **H-L:**8.510 ***p*** = 0.385 **SP** = 80.4 **SE** = 69.8 **Ov** = 75.8
Years of schooling	0.006	0.043	0.017	0.896	1.006	0.925–1.094		−0.022	0.060	0.140	0.709	0.978	1.194–1.099	
Self-report SCCs	−0.030	0.062	0.227	0.634	0.971	0.860–1.096		0.356	0.091	15.188	<0.001**	1.428	1.428–1.194	
Informant-reported SCCs	0.154	0.060	6.707	0.010*	1.167	1.038–1.311		0.045	0.077	0.339	0.560	1.046	0.899–1.217	
Depressive symptomatology	0.142	0.076	3.469	0.063	1.153	0.993–1.338		0.193	0.095	4.089	0.043*	1.212	1.006–1.461	
**(2) CU to MCI**	No cases, **STABLE CU:** 86; Cases, **progression to MCI**: 12							No cases, **STABLE CU:** 56; Cases, **progressed to MCI**: 11						
Age	0.032	0.035	0.852	0.356	1.033	0.965–1.106	**N of *R***^**2**^: 0.115 **H-L:** 4.000 ***p*** = 0.857 **SP** = 100 **SE** = 8.3 **Ov** = 88.8	0.091	0.045	4.168	0.041*	1.096	1.004–1.196	**N of *R***^**2**^: 0.224 **H-L:** 4.371 ***p*** = 0.822 **SP** = 98.2 **SE** = 18.2 **Ov** = 85.1
Years of schooling	−0.051	0.074	0.466	0.495	0.951	0.822–1.099		−0.019	0.102	0.036	0.849	0.981	0.803–1.198	
Self-reported SCCs	−0.148	0.101	2.140	0.144	0.863	0.708–1.051		0.042	0.129	0.107	0.743	1.043	0.810–1.344	
Informant-reported SCCs	0.087	0.088	0.973	**0.**324	1.091	0.918–1.297		0.132	0.096	1.865	0.172	1.141	0.944–1.378	
Depressive symptomatology	0.132	0.140	0.897	**0.**344	1.142	0.868–1.502		0.122	0.146	0.702	0.402	1.130	0.849–1.505	
**(3) SCD to MCI**	No cases, **STABLE SCD**: 81; Cases, **progressed to MCI:** 11							No cases, **STABLE SCD**: 59; Cases, **progressed to MCI**: 8						
Age	0.066	0.050	1.755	0.185	1.068	0.969–1.178	**N of *R***^**2**^: 0.186 **H-L:** 5.890 ***p*** = 0.660 **SP** = 98.8 **SE** = 9.1 **Ov** = 88.2	0.188	0.092	4.616	0.032*	1.219	1.018–1.460	**N of *R***^**2**^: 0.566 **H-L:** 11.366 ***p*** = 0.182 **SP** = 98.3 **SE** = 50.0 **Ov** = 92.5
Years of schooling	−0.237	0.117	4.086	0.043*	0.789	0.627–0.993		−0.175	0.215	0.662	0.416	0.839	0.550–1.280	
Self-reported SCCs	−0.048	0.095	0.251	0.617	0.954	0.791–1.149		−0.312	0.165	3.562	0.059	0.738	0.530–1.012	
Informant-reported SCCs	0.068	0.080	0.724	**0.**395	1.070	0.915–1.251		0.473	0.199	5.672	0.017*	1.605	1.087–2.369	
Depressive symptomatology	0.068	0.099	0.476	0.490	1.071	0.881–1.301		0.592	0.261	5.138	0.023*	1.808	1.083–3.016	
**(4) SCD to dementia**	No cases, **STABLE SCD**: 81; Cases, **converted to dementia:** 9							No cases, **STABLE SCD**: 59; Cases, **converted to dementia**: 7						
Age	0.158	0.073	4.686	0.030*	1.171	0.015–1.350	**N of *R***^**2**^: 0.293 **H-L:** 2.129 ***p*** = 0.977 **SP** = 98.8 **SE** = 0.0 **Ov** = 88.9	0.184	0.087	4.512	0.034*	1.202	1.014–1.425	**N of *R***^**2**^: 0.478 **H-L:** 7.019 ***p*** = 0.427 **SP** = 98.3 **SE** = 42.9 **Ov** = 92.4
Years of schooling	−0.153	0.097	2.476	0.116	0.858	0.710–1.038		−0.171	0.143	1.442	0.230	0.843	0.637–1.114	
Self-reported SCCs	−0.201	0.133	2.277	0.131	0.818	0.630–1.062		−0.149	0.139	1.148	0.284	0.861	0.656–1.132	
Informant-reported SCCs	0.191	0.119	2.574	0.109	1.210	0.959–1.527		0.372	0.146	6.479	0.011*	1.451	1.086–1.933	
Depressive symptomatology	−0.323	0.191	2.869	0.090	0.724	0.498–1.052		−0.148	0.274	0.291	0.589	0.863	0.504–1.475	
**(5) MCI to dementia**	No cases, **STABLE MCI**: 42; Cases, **converted to dementia**: 22							No cases, **STABLE MCI**: 29; Cases, **converted to dementia**: 6						
Age	0.050	0.047	1.151	0.283	1.051	0.960–1.152	**N of *R***^**2**^: 0.226 **H-L:** 6.715 ***p*** = 0.568 **SP** = 83.3 **SE** = 45.5 **Ov** = 70.3	0.095	0.108	0.778	0.378	1.100	0.891–1.358	**N of *R***^**2**^: 0.476 **H-L:**8.293 ***p*** = 0.307 **SP** = 96.6 **SE** = 50.0 **Ov** = 88.6
Years of schooling	0.031	0.065	0.224	0.636	1.032	0.907–1.173		−0.023	0.200	0.014	0.907	0.977	0.660–1.447	
Self-reported SCCs	−0.176	0.087	4.064	0.044*	0.839	0.707–0.995		−0.440	0.221	3.965	0.046*	0.644	0.418–0.993	
Informant-reported SCCs	0.141	0.083	2.893	0.089	1.152	0.979–1.355		0.259	0.189	1.884	0.179	1.296	0.895–1.876	
Depressive symptomatology	0.013	0.125	0.011	0.915	1.013	0.794–1.294		0.371	0.219	2.882	0.090	1.450	0.994–2.226	

FU, follow-up; CU, cognitively unimpaired; SCD, subjective cognitive decline; MCI, mild cognitive impairment; *N* of *R*^2^, Nagelkerke R square; H-L, Hosmer and Lemeshow test; SP, specificity; SE, sensitivity; Ov, overall percentage; binary regression logistic. Significance level *p < 0.05; **p < 0.001.

### 3.2 Predicting progression in the SCD group

In the SCD group, progression to MCI was significantly associated with fewer years of schooling (*β* = −0.237, *SE* = 0.117, *p* = 0.043, *OR* = 0.789, *CI* = 0.627–993), for early prediction, and with increasing age (*β* = 0.188, *SE* = 0.092, *p* = 0.032, *OR* = 1.219, *CI* = 1.018–1.460), higher informant complaints (*β* = 0.473, *SE* = 0.199, *p* = 0.017, *OR* = 1.605, *CI* = 1.087–2.369) and higher depressive symptomatology (*β* = 0.592, *SE* = 0.261, *p* = 0.023, *OR* = 1.808, *CI* = 1.083–3.016), for late prediction (see Table 3). The model fits were acceptable, and the Sensitivity of classification was higher for late prediction (50.0) than for early prediction (9.1).

Conversion to dementia was significantly associated with increasing age (*β* = 0.158, *SE* = 0.073, *p* = 0.030, *OR* = 1.171, *CI* = 0.015–1.350), for early prediction, and with increasing age (*β* = 0.184, *SE* = 0.087, *p* = 0.034, *OR* = 1.202, *CI* = 1.014–1.425) and higher informant-reported SCCs (*β* = 0.372, *SE* = 0.146, *p* = 0.011, *OR* = 1.451, *CI* = 1.086–1933), for late prediction. The Sensitivity of classification was higher for late (42.9) than for early prediction (0.0).

### 3.3 Predicting conversion in the MCI group

In the MCI group, conversion to dementia was only significantly associated with fewer patient-reported SCCs, for early prediction (*β* = −0.176, *SE* = 0.087, *p* = 0.044, *OR* = 0.839, *CI* = 0.707–0.995) and late prediction (*β* = −0.440, *SE* = 0.221, *p* = 0.046, *OR* = 0.644, *CI* = 0.418–0.993). Classification values were higher for late prediction (Specificity =96.6, Sensitivity = 50.0, Overall percentage = 88.6) than for early prediction (Specificity =83.3, Sensitivity = 45.5, Overall percentage = 70.3) (see Table 3).

### 3.4 Predicting reversion in the SCD group

In the SCD group, reversion to CU was significantly associated only with fewer patient-reported SCCs (*β* = −0.440, *SE* = 0.162, *p* = 0.007, *OR* = 0.644, *CI* = 0.469–0.884) only for late prediction, with a low Sensitivity of classification (10.0) (Table 4).

**Table 4:** Prediction of reversion to cognitively unimpaired (CU) and/or subjective cognitive decline (SCD).

	EARLY PREDICTION Time in months: M 58.87; SD 8.26 –From baseline to the 3rd FU–							LATE PREDICTION Time in months: M 21.60; SD 7.36 –From the 2nd FU the 3rd FU –						
(1) SCD to CU	No cases, **STABLE SCD**: 81; Cases, **reverted to CU**: 10							No cases, **STABLE SCD**: 59; Cases, **reverted to CU**: 10						
	***β***	***S.E.***	***Wald***	***p***	***OR***	***95% CI***	***Models***	***β***	***S.E.***	***Wald***	***p***	***OR***	***95%CI***	***Models***
Age	−0.091	0.049	3.469	0.063	0.913	0.830–1.005	**N of *R***^**2**^: 0.212 **H-L:** 12.204 ***p*** = 0.142 **SP** = 100 **SE** = 20.0 **Ov** = 91.2	−0.071	0.054	1.743	0.187	0.931	0.838–1.035	**N of *R***^**2**^: 0.356 **H-L:** 4.942 ***p*** = 0.764 **SP** = 94.9 **SE** = 10.0 **Ov** = 82.6
Years of schooling	0.139	0.086	2.603	0.107	1.149	0.971–1.361		0.105	0.104	1.005	0.316	1.110	0.905–1.362	
Self-reported SCCs	−0.179	0.138	1.680	0.195	0.837	0.639–1.096		−0.440	0.162	7.399	0.007*	0.644	0.469–0.884	
Informant-reported SCCs	0.031	0.097	0.100	**0.**752	1.031	0.853–1.246		−0.122	0.116	1.098	0.295	0.885	0.705–1.112	
Depressive symptomatology	0.137	0.123	1.233	0.267	1.147	0.901–1.460		0.322	0.194	2.755	0.097	1.380	0.943–2.018	
(2) MCI to CU and/or SCD	No cases, **STABLE MCI:** 42; Cases, **reverted to CU and/or SCD**: 17							No cases, **STABLE MCI:** 29; Cases, **reverted to CU and/or SCD**: 17						
Age	−0.136	0.052	6.784	0.009*	0.873	0.787–0.967	**N of *R***^**2**^: 0.268 **H-L:** 7.907 ***p*** = 0.443 **SP** = 90.5 **SE** = 29.4 **Ov** = 72.9	−0.110	0.064	2.974	0.085	0.896	0.791–1.015	**N of *R***^**2**^: 0.524 **H-L:**6.873 ***p*** = 0.442 **SP** = 89.7 **SE** = 64.7 **Ov** = 80.4
Years of schooling	−0.158	0.111	2.028	0.154	0.854	0.688–1.061		−0.318	0.151	4.467	0.035*	0.728	0.542–0.977	
Self-reported SCCs	−0.122	0.092	1.762	0.184	0.885	0.739–1.060		−0.362	0.176	4.250	0.039*	0.696	0.494–0.982	
Informant-reported SCCs	−0.051	0.094	0.295	0.587	0.950	0.790–1.143		−0.155	0.155	0.995	0.319	0.857	0.632–1.161	
Depressive symptomatology	0.057	0.137	0.172	0.679	1.059	0.809–1.386		0.316	0.160	3.925	0.048*	1.372	1.372–1.876	

FU, follow-up; CU, cognitively unimpaired; SCD, subjective cognitive decline; MCI, mild cognitive impairment; *N* of *R*^2^, Nagelkerke R square; H-L, Hosmer and Lemeshow test; SP, specificity; SE, senstivity; Ov, overall percentage; binary regression logistic. Significance level *p < 0.05; **p < 0.001.

### 3.5 Predicting reversion in the MCI group

In the MCI group, reversion to CU and/or SCD was significantly associated with fewer years of education (*β* = −0.318, *SE* = 0.151, *p* = 0.035, *OR* = 0.728, *CI* = 0.542–0.977), fewer self-reported SCCs (*β* = −0.362, *SE* = 0.176, *p* = 0.039, *OR* = 0.696, *CI* = 0.494–0.981) and higher depressive symptomatology (*β* = 0.316, *SE* = 0.160, *p* = 0.048, *OR* = 1.372, *CI* = 1.372–1.876), only for later prediction (see Table 4). Classification values were Specificity =89.7, Sensitivity = 64.7, Overall percentage = 80.4.

## 4 Discussion

The current research aimed to gather new evidence on the role of dyadic cognitive reports on the cognitive decline along the continuum from CU to dementia. The study findings showed that the predictive value of self- and informant-reported cognitive complaints and depressive symptoms varies depending on the type of transition (i.e., diagnostic group of origin and outcome) and on the time at which the prediction is made (i.e., early, late). They also indicated the influence of depressive symptoms, age and education at the different stages and times of prediction.

For CU participants, informant-reported cognitive complaints at baseline were an early predictor of progression to SCD. At later dates, self-reports emerged along with depressive symptomatology, already shown to be close to significance in early prediction, as significant predictors of progression to SCD. The findings support the importance of informant-reported measures for the early prediction of progression to SCD in CU participants. Self-reported SCCs experienced at later dates only enable late prediction of SCD at stages close to this state; these reports are characterized by an increase in SCCs that are worrying to participants because they seem to be aware of the subtle cognitive and neuropsychiatric changes (Jessen et al., 2014; Jack et al., 2018). This is consistent with a previous finding indicating that at the beginning of the deterioration process participants reported more SCCs than informants (Numbers et al., 2023). Our findings also indicate the important role of an increase in depressive symptoms (Comijs et al., 2002; Burmester et al., 2016; Markova et al., 2017; Tandetnik et al., 2017; Kim and Lee, 2022), especially when the change from CU to SCD is imminent (i.e., late prediction). However, complaints failed to predict early or late progression to MCI in CU participants, supporting the negligible role of mild complaints (below the 5th percentile typical of CU) in estimating the risk of objective cognitive impairment that characterizes the MCI stage (Pereiro et al., 2021). In this case only increasing age (indicated by the positive beta value, Table 3) predicted the emergence of objective cognitive impairment. Our findings shed new light on the role of self- and informant-reported complaints at the initial stage of the continuum. Previous research involving CU participants indicated that both self- and informant-reported measures predict the risk of cognitive decline (Nosheny et al., 2022). However, our findings suggest that the role of one or the other depends on the stage of progression and on the time at which the prediction is made (early or late): informant-reported complaints were significant for early prediction of the progression to SCD, and the self-reported complaints were significant at later stages (late prediction), closer in time to the change of stage. However, neither self-reported or informant-reported complaints reached the level of significance required to predict the risk of progression from CU to MCI.

Regarding progression from SCD to MCI, we found education to be the only significant predictor in early prediction. A low level of education (fewer years of schooling), indicated by a negative beta value (Table 3), can predict progression to MCI. We suggest that using the level of education as a proxy for cognitive reserve may prevent identification of cognitive deterioration (Lojo-Seoane et al., 2018) at this early stage of cognitive decline. Later, when progression to MCI is closer (late prediction) informant-reports and depressive symptomatology emerged as significant predictors of progression. The present findings are consistent with those of Dolcet-Negre et al. (2023), who found that depressive symptoms were predictors of progression from SCD to MCI and/or dementia. On the other hand, only informant reports and not participant reports, expressed closer to the time of progression (late prediction), were able to predict progression to MCI and also to dementia. A higher level of informant-reported SCCs, indicated by a positive beta value (Table 3), appears critical for predicting late progression from SCD to MCI and dementia. However, a lower level of participant-reported SCCs (negative beta values) for this late progression was almost significant (*p* = 0.059). These results are consistent with a previous report of a negative discrepancy between patient-reported and informant-reported SCCs in MCI, with lower scores from patients than from informants, with the negative discrepancy interpreted as a decreased awareness of MCI (Ryu et al., 2020). Our findings also add new data about the SCD stage which was not included in the review by Nosheny et al. (2022), and they may provide further evidence for the role of the dyadic reports at the first steps of the continuum. At this stage, informant reports, but not self reports, estimate progression to MCI and to dementia. The findings are also consistent with those of other studies that suggest that the informant-reported SCCs are successful predictors of biomarkers of preclinical AD and incident dementia (Numbers et al., 2023) and increased global amyloid load over time (Kuhn et al., 2021). The former study consistently showed that both patient- and informant-reported SCCs at baseline were separately able to predict incident dementia for SCD patients, but only the informant successfully predicted longitudinal change (Numbers et al., 2023).

Informants seem to identify cognitive decline better for transition from SCD to MCI, and for transition from SCD to dementia; however, patients make more valid estimates at the early asymptomatic stages, e.g., from CU to SCD. Our complementary results on the patients who reverted from SCD to CU and from MCI to SCD and CU (Table 4) are also consistent with this statement. In the clinical reversion from SCD to CU, the self-reported SCCs was the only significant variable in the late prediction of improvement in cognitive status. Self-reported SCCs also predicted, together with education and depressive symptomatology, late reversion from MCI to SCD and CU. The beta values in the self-reports were always negative, indicating that low scores in complaints are those that predict reversal, i.e., improvement in cognitive status. The findings can be interpreted considering the theory of Awareness Cognitive Decline. Thus, comparison of the negative beta values for late reversion and the positive values shown for the same variable (self-reports) in late progression from CU to SCD (Table 3) shows that the decrease in complaints that predicts improvement and the increase in complaints that predicts deterioration both indicate that the patient still has a high level of awareness about their condition at these early stages. This high level of awareness could also be related to the significant role of depressive symptomatology, which yielded positive beta values both in reversion and in progression, indicating that participants were aware of and worried about their cognitive status. The statistical significance of high depressive symptomatology (with a positive beta value) is also evident in the late progression from SCD to MCI, which again suggests that patients worry about subsequent cognitive deterioration. By contrast, the lack of importance of depression measures in predicting late conversion from SCD and MCI to dementia may be consistent with the interference of anosognosia on the ability to report depressive symptoms (Munro et al., 2022).

Surprisingly, our results indicate that in the progression from MCI to dementia, only self-reported SCCs were statistically significant, both for early prediction and late prediction. However, the beta values were negative in both cases, which suggests that the prediction is determined by the participants’ low scores on the complaint’s questionnaire. This result could also be interpreted from the point of view of the Awareness of Cognitive Decline (ACD) concept as MCI is characterized by low awareness of cognitive decline (Ryu et al., 2020) and as a decrease in awareness can increase the risk of and/or predict the progression to dementia (Munro et al., 2018; Cacciamani et al., 2020; Vannini et al., 2020).

Several limitations of our study must be considered. First, the predictive value of self-reports and informant-reports could be modulated by some personal (e.g., personality traits, anxiety) or relational (e.g., participant-informant relationship, frequency of social contact) factors not considered in this study and that should be evaluated in the future.

Second, the design of the study of the progression from the different stages of the cognitive continuum and at the different time (early and late) imposed several constraints: (A) the use of binary analysis instead of continuous analysis because we had to establish groups according to the diagnostic criteria characterizing each stage. Within these groups, we defined progression in order to determine the predictive value of SCCs for participants who progress or were stable from baseline to the end (early prediction) and for those who progress or were stable from the second follow-up to the end (late prediction). Although we believe that this design is clinically relevant and can serve to establish the diagnostic value of SCCs for each diagnostic group and for each stage of progression, it required a larger number of groups, and therefore reduced the size of each and increased the comparisons required. (B) Consequently, the low number of progressions identified in some diagnostic groups and at some of the moments reduced the number of cases (those who progress) and influenced the results of the logistic binary regression, which in these cases should be considered with caution. We expect that in the near future our ongoing longitudinal project with several cohorts will include larger samples and a greater number of diagnostic transitions. (C) The inclusion of only five predictive variables in the logistic regression models (age, education, depression, self- and informant-reported SCCs) led to low or moderate fits of the data to the models, which only explained up to 56% of the variance. However, considering that progression of cognitive decline is determined by many sociocultural, physical health, emotional, and biological variables, selection of the five predictor variables used was not intended to produce very accurate prediction of progression, but rather to determine the extent to which self- and informant-reported SCCs can predict the progression of cognitive decline. The variables age, years of education and depressive symptoms were also considered given their possible relationship with increased cognitive impairment. (D) Similarly, the sensitivity values were low and ranged between 42.9 and 69.8 in the best models, which indicates that although the specificity values (always greater than 80.0) and overall classification (greater than 67.0) were acceptable, the accuracy of the models that included the five variables was too low to predict progression.

Finally, we identified progression of cognitive decline in all participants considering their corresponding diagnosis at the end of the evaluation process. Thus, for some participants the progression took place at the first follow-up, and for others it took place at the second or third follow-up. We are aware that the progression time differs across participants and that this has implications for the rate of progression. However, considering the exact time of progression for each participant would imply establishing very large numbers of groups, which would increase the complexity of data analysis and interpretation. In the near future new continuous analyses with larger samples may enable inclusion of speed of progression in the research design.

## 5 Conclusion

The study provides new data that help clarify the role of SCCs reported by patients and their informants on the progression within the continuum of cognitive impairment, considering the initial stages and two moments in which these SCCs occur, distant from and close to the final outcome. A late increase in self-reported complaints make valid estimates to predict subjective decline from CU. Decrease in early and late self-reports also successfully predict dementia from prodromic stage. As deterioration increases, i.e., when the SCD is progressing to MCI or dementia, the informant-reported SCCs are the best predictors of progression. Depressive symptomatology was also a predictor of the progression from both CU to SCD and from SCD to MCI. Only late decrease in self-reported complaints predict reversion from prodromic and pre-symptomatic stages.

Although our study was based on the separate analysis of patient- and informant-reported SCCs, the findings can also be interpreted using the Awareness Cognitive Decline theory. The predictive and successive role of self-reported and informant-reported SCCs on the progression of deterioration between the CU, SCD and MCI stages may be due to a gradual decline in cognitive awareness. New longitudinal studies with large samples are necessary to confirm the predictive role of SCCs reported by patients and informants at the different stages of cognitive deterioration, while also considering the evolution of cognitive awareness.

## Data availability statement

The raw data supporting the conclusions of this article will be made available by the authors, without undue reservation.

## Ethics statement

The studies involving humans were approved by Galician Autonomous Committee on Research Ethics (Xunta de Galicia, Spain). The studies were conducted in accordance with the local legislation and institutional requirements. Written informed consent for participation in this study was provided by the participants’ legal guardians/next of kin. Written informed consent was obtained from the individual(s) for the publication of any potentially identifiable images or data included in this article.

## Author contributions

LP-B: Data curation, Methodology, Writing – original draft, Investigation. AF-L: Data curation, Methodology. AN-V: Data curation, Investigation. CL-S: Data curation, Methodology. MC-M: Data curation, Methodology. FF-F: Data curation, Investigation. OJ-R: Methodology, Writing – review & editing. AP: Methodology, Data curation, Writing – original draft.
